# Supplementary material for: Publication Bias in Meta-Analysis: Confidence Intervals for Rosenthal's Fail-Safe Number
Source: Int Sch Res Notices. 2014 Dec 3;2014:825383. doi: 10.1155/2014/825383 (PMC4897051; doi:10.1155/2014/825383)
Supplement: Supplementary file 1 — The Supplementary Materials contain the simulation code used in R and the Z-values from the meta-analyses used as examples in Section 5. [file 825383.f1.docx]

# **Supplementary Materials**

# **Code for Simulations in R**

**### Examining the Standard Normal distribution mu and sigma-square values and drawing Zi from all four distributions###**

**#######################**

R=10000 ; B=1000

## R is the number of simulations, B is the number of bootstrap resamples

rosent1=matrix(nrow=R,ncol=4)

## here we will store the Rosenthal's values for the 4 sample sizes

v1=array(dim=c(R,5,4))

## the variances will be stored here

mat1=mat2=mat3=mat4=mat5=array(dim=c(R,2,4))

## the confidence intervals will be stored here

za=qnorm(0.95) ; n=lam=c(5,15,30,50) ; f=(n-1)/n

## f is used to get the unbiased variance estimator

m=0 ; s=1

## parameters of the normal distribution

fixed1=(n^2*m^2+n*s)/za^2-n

## real values of Rosenthal's as fixed

random1=(lam^2*m^2+lam*(m^2+s))/za^2-lam

## real values of Rosenthal's as random

**coverage1**=matrix(nrow=4,ncol=6)

## the coverages will be stored here

set.seed(123456)

## seed number

for (k in 1:4) {

for (i in 1:R) {

**z=rnorm(n[k])**

**## random values of z-statistics are generated**

**## When we draw from the half normal we use z=abs(rnorm(n[k],0,1)); when we draw from the skew normal distribution we use z=rsn(n[k],xi=0,omega=1,alpha=-0.5773503) or z=rsn(n[k],xi=0,omega=1,alpha=0.5773503) for negative and positive skewness respectively ###**

**###############**

rosent1[i,k]=(sum(z)/za)^2-n[k]

## Rosenthal's value

m1=0 ; s1=1

v1[i,1,k]=2*n[k]^2*s1*(2*n[k]*m1^2+s1)/za^4

## distributional variance of the fixed studies

m2=mean(z) ; s2=f[k]*var(z)

v1[i,2,k]=2*n[k]^2*s2*(2*n[k]*m2^2+s2)/za^4

## moments variance of the fixed studies

v1[i,3,k]=( (4*lam[k]^3+6*lam[k]^2+lam[k])*m1^4+(4*lam[k]^3+16*lam[k]^2+6*lam[k])*m1^2*s1+

(2*lam[k]^2+3*lam[k])*s1^2 )/za^4- 2*( (2*lam[k]^2+lam[k])*m1^2+lam[k]*s1 )/za^2+lam[k]

## distributional variance of the random studies

v1[i,4,k]=( (4*lam[k]^3+6*lam[k]^2+lam[k])*m2^4+(4*lam[k]^3+16*lam[k]^2+6*lam[k])*m2^2*s2+

(2*lam[k]^2+3*lam[k])*s2^2 )/za^4- 2*( (2*lam[k]^2+lam[k])*m2^2+lam[k]*s2 )/za^2+lam[k]

## moments variance of the random studies

## then is the bootstrap case

t=rep(0,B)

for (j in 1:B) {

nu=sample(1:n[k],n[k],replace=T)

t[j]=(sum(z[nu])/za)^2-n[k] }

v1[i,5,k]=var(t)

mat1[i,,k]=c(rosent1[i,k]-1.96*sqrt(v1[i,1,k]),rosent1[i,k]+1.96*sqrt(v1[i,1,k]))

mat2[i,,k]=c(rosent1[i,k]-1.96*sqrt(v1[i,2,k]),rosent1[i,k]+1.96*sqrt(v1[i,2,k]))

mat3[i,,k]=c(rosent1[i,k]-1.96*sqrt(v1[i,3,k]),rosent1[i,k]+1.96*sqrt(v1[i,3,k]))

mat4[i,,k]=c(rosent1[i,k]-1.96*sqrt(v1[i,4,k]),rosent1[i,k]+1.96*sqrt(v1[i,4,k]))

mat5[i,,k]=c(rosent1[i,k]-1.96*sqrt(v1[i,5,k]),rosent1[i,k]+1.96*sqrt(v1[i,5,k])) } }

for (l in 1:4) coverage1[l,1]=1-(sum(mat1[,1,l]>fixed1[l])/R+sum(mat1[,2,l]<fixed1[l])/R)

for (l in 1:4) coverage1[l,2]=1-(sum(mat2[,1,l]>fixed1[l])/R+sum(mat2[,2,l]<fixed1[l])/R)

for (l in 1:4) coverage1[l,3]=1-(sum(mat3[,1,l]>random1[l])/R+sum(mat3[,2,l]<random1[l])/R)

for (l in 1:4) coverage1[l,4]=1-(sum(mat4[,1,l]>random1[l])/R+sum(mat4[,2,l]<random1[l])/R)

for (l in 1:4) coverage1[l,5]=1-(sum(mat5[,1,l]>fixed1[l])/R+sum(mat5[,2,l]<fixed1[l])/R)

for (l in 1:4) coverage1[l,6]=1-(sum(mat5[,1,l]>random1[l])/R+sum(mat5[,2,l]<random1[l])/R)

colnames(coverage1)=c('Dist_fixed','Mom_fixed','Dist_random','Mom_random',

'Boot_fixed', 'Boot_random')

rownames(coverage1)=c('n=5','n=15','n=30','n=50')

**### Examining the Half Normal distribution mu and sigma-square values and drawing Zi from all four distributions###**

**#####################**

R=10000 ; B=1000

## R is the number of simulations, B is the number of bootstrap resamples

rosent2=matrix(nrow=R,ncol=4)

## here we will store the Rosenthal's values for the 4 sample sizes

v2=array(dim=c(R,5,4))

## the variances will be stored here

mat6=mat7=mat8=mat9=mat10=array(dim=c(R,2,4))

## the confidence intervals will be stored here

za=qnorm(0.95) ; n=lam=c(5,15,30,50) ; f=(n-1)/n

## f is used to get the unbiased variance estimator

m=sqrt(2/pi) ; s=1-2/pi

## parameters of the half normal distribution

fixed2=(n^2*m^2+n*s)/za^2-n

## real values of Rosenthal's as fixed

random2=(lam^2*m^2+lam*(m^2+s))/za^2-lam

## real values of Rosenthal's as random

**coverage2**=matrix(nrow=4,ncol=6)

## the coverages will be stored here

set.seed(123456)

## seed number

for (k in 1:4) {

for (i in 1:R) {

**z=rnorm(n[k])**

**## random values of z-statistics are generated**

**## When we draw from the half normal we use z=abs(rnorm(n[k],0,1)); when we draw from the skew normal distribution we use z=rsn(n[k],xi=0,omega=1,alpha=-0.5773503) or z=rsn(n[k],xi=0,omega=1,alpha=0.5773503) for negative and positive skewness respectively ###**

**###############**

rosent2[i,k]=(sum(z)/za)^2-n[k]

## Rosenthal's value

m1=sqrt(2/pi) ; s1=1-2/pi

v2[i,1,k]=2*n[k]^2*s1*(2*n[k]*m1^2+s1)/za^4

## distributional variance of the fixed studies

m2=mean(z) ; s2=f[k]*var(z)

v2[i,2,k]=2*n[k]^2*s2*(2*n[k]*m2^2+s2)/za^4

## moments variance of the fixed studies

v2[i,3,k]=( (4*lam[k]^3+6*lam[k]^2+lam[k])*m1^4+(4*lam[k]^3+16*lam[k]^2+6*lam[k])*m1^2*s1+

(2*lam[k]^2+3*lam[k])*s1^2 )/za^4-2*( (2*lam[k]^2+lam[k])*m1^2+lam[k]*s1 )/za^2+lam[k]

## distributional variance of the random studies

v2[i,4,k]=( (4*lam[k]^3+6*lam[k]^2+lam[k])*m2^4+(4*lam[k]^3+16*lam[k]^2+6*lam[k])*m2^2*s2+

(2*lam[k]^2+3*lam[k])*s2^2 )/za^4-2*( (2*lam[k]^2+lam[k])*m2^2+lam[k]*s2 )/za^2+lam[k]

## moments variance of the random studies

## then is the bootstrap case

t=rep(0,B)

for (j in 1:B) {

nu=sample(1:n[k],n[k],replace=T)

t[j]=(sum(z[nu])/za)^2-n[k] }

v2[i,5,k]=var(t)

mat6[i,,k]=c(rosent2[i,k]-1.96*sqrt(v2[i,1,k]),rosent2[i,k]+1.96*sqrt(v2[i,1,k]))

mat7[i,,k]=c(rosent2[i,k]-1.96*sqrt(v2[i,2,k]),rosent2[i,k]+1.96*sqrt(v2[i,2,k]))

mat8[i,,k]=c(rosent2[i,k]-1.96*sqrt(v2[i,3,k]),rosent2[i,k]+1.96*sqrt(v2[i,3,k]))

mat9[i,,k]=c(rosent2[i,k]-1.96*sqrt(v2[i,4,k]),rosent2[i,k]+1.96*sqrt(v2[i,4,k]))

mat10[i,,k]=c(rosent2[i,k]-1.96*sqrt(v2[i,5,k]),rosent2[i,k]+1.96*sqrt(v2[i,5,k])) } }

for (l in 1:4) coverage2[l,1]=1-(sum(mat6[,1,l]>fixed2[l])/R+sum(mat6[,2,l]<fixed2[l])/R)

for (l in 1:4) coverage2[l,2]=1-(sum(mat7[,1,l]>fixed2[l])/R+sum(mat7[,2,l]<fixed2[l])/R)

for (l in 1:4) coverage2[l,3]=1-(sum(mat8[,1,l]>random2[l])/R+sum(mat8[,2,l]<random2[l])/R)

for (l in 1:4) coverage2[l,4]=1-(sum(mat9[,1,l]>random2[l])/R+sum(mat9[,2,l]<random2[l])/R)

for (l in 1:4) coverage2[l,5]=1-(sum(mat10[,1,l]>fixed2[l])/R+sum(mat10[,2,l]<fixed2[l])/R)

for (l in 1:4) coverage2[l,6]=1-(sum(mat10[,1,l]>random2[l])/R+sum(mat10[,2,l]<random2[l])/R)

colnames(coverage2)=c('Dist_fixed','Mom_fixed','Dist_random','Mom_random',

'Boot_fixed', 'Boot_random')

rownames(coverage2)=c('n=5','n=15','n=30','n=50')

**### Examining the Skew Normal distribution (negative skewness) mu and sigma-square values and drawing Zi from all four distributions###**

**########################**

R=10000 ; B=1000

## R is the number of simulations, B is the number of bootstrap resamples

rosent3=matrix(nrow=R,ncol=4)

## here we will store the Rosenthal's values for the 4 sample sizes

v3=array(dim=c(R,5,4))

## the variances will be stored here

mat11=mat12=mat13=mat14=mat15=array(dim=c(R,2,4))

## the confidence intervals will be stored here

za=qnorm(0.95) ; n=lam=c(5,15,30,50) ; f=(n-1)/n

## f is used to get the unbiased variance estimator

m=-sqrt(1/(2*pi)) ; s=1-1/(2*pi)

fixed3=(n^2*m^2+n*s)/za^2-n

## real values of Rosenthal's as fixed

random3=(lam^2*m^2+lam*(m^2+s))/za^2-lam

## real values of Rosenthal's as random

**coverage3**=matrix(nrow=4,ncol=6)

set.seed(123456)

## seed number

for (k in 1:4) {

for (i in 1:R) {

**z=rnorm(n[k])**

**## random values of z-statistics are generated**

**## When we draw from the half normal we use z=abs(rnorm(n[k],0,1)); when we draw from the skew normal distribution we use z=rsn(n[k],xi=0,omega=1,alpha=-0.5773503) or z=rsn(n[k],xi=0,omega=1,alpha=0.5773503) for negative and positive skewness respectively ###**

**###############**

rosent3[i,k]=(sum(z)/za)^2-n[k]

## Rosenthal's value

m1=-sqrt(2/pi) ; s1=1-2/pi

v3[i,1,k]=2*n[k]^2*s1*(2*n[k]*m1^2+s1)/za^4

## distributional variance of the fixed studies

m2=mean(z) ; s2=f[k]*var(z)

v3[i,2,k]=2*n[k]^2*s2*(2*n[k]*m2^2+s2)/za^4

## distributional variance of the random studies

v3[i,3,k]=( (4*lam[k]^3+6*lam[k]^2+lam[k])*m1^4+(4*lam[k]^3+16*lam[k]^2+6*lam[k])*m1^2*s1+

(2*lam[k]^2+3*lam[k])*s1^2 )/za^4-2*( (2*lam[k]^2+lam[k])*m1^2+lam[k]*s1 )/za^2+lam[k]

## moments variance of the fixed studies

v3[i,4,k]=( (4*lam[k]^3+6*lam[k]^2+lam[k])*m2^4+(4*lam[k]^3+16*lam[k]^2+6*lam[k])*m2^2*s2+

(2*lam[k]^2+3*lam[k])*s2^2 )/za^4-2*( (2*lam[k]^2+lam[k])*m2^2+lam[k]*s2 )/za^2+lam[k]

## moments variance of the random studies

## then is the bootstrap case

t=rep(0,B)

for (j in 1:B) {

nu=sample(1:n[k],n[k],replace=T)

t[j]=(sum(z[nu])/za)^2-n[k] }

v3[i,5,k]=var(t)

mat11[i,,k]=c(rosent3[i,k]-1.96*sqrt(v3[i,1,k]),rosent3[i,k]+1.96*sqrt(v3[i,1,k]))

mat12[i,,k]=c(rosent3[i,k]-1.96*sqrt(v3[i,2,k]),rosent3[i,k]+1.96*sqrt(v3[i,2,k]))

mat13[i,,k]=c(rosent3[i,k]-1.96*sqrt(v3[i,3,k]),rosent3[i,k]+1.96*sqrt(v3[i,3,k]))

mat14[i,,k]=c(rosent3[i,k]-1.96*sqrt(v3[i,4,k]),rosent3[i,k]+1.96*sqrt(v3[i,4,k]))

mat15[i,,k]=c(rosent3[i,k]-1.96*sqrt(v3[i,5,k]),rosent3[i,k]+1.96*sqrt(v3[i,5,k])) } }

for (l in 1:4) coverage3[l,1]=1-(sum(mat11[,1,l]>fixed3[l])/R+sum(mat11[,2,l]<fixed3[l])/R)

for (l in 1:4) coverage3[l,2]=1-(sum(mat12[,1,l]>fixed3[l])/R+sum(mat12[,2,l]<fixed3[l])/R)

for (l in 1:4) coverage3[l,3]=1-(sum(mat13[,1,l]>random3[l])/R+sum(mat13[,2,l]<random3[l])/R)

for (l in 1:4) coverage3[l,4]=1-(sum(mat14[,1,l]>random3[l])/R+sum(mat14[,2,l]<random3[l])/R)

for (l in 1:4) coverage3[l,5]=1-(sum(mat15[,1,l]>fixed3[l])/R+sum(mat15[,2,l]<fixed3[l])/R)

for (l in 1:4) coverage3[l,6]=1-(sum(mat15[,1,l]>random3[l])/R+sum(mat15[,2,l]<random3[l])/R)

colnames(coverage3)=c('Dist_fixed','Mom_fixed','Dist_random','Mom_random',

'Boot_fixed', 'Boot_random')

rownames(coverage3)=c('n=5','n=15','n=30','n=50')

**### Examining the Skew Normal distribution (positive skewness) mu and sigma-square values and drawing Zi from all four distributions###**

#######################

R=10000 ; B=1000

## R is the number of simulations, B is the number of bootstrap resamples

rosent4=matrix(nrow=R,ncol=4)

## here we will store the Rosenthal's values for the 4 sample sizes

v4=array(dim=c(R,5,4))

## the variances will be stored here

mat16=mat17=mat18=mat19=mat20=array(dim=c(R,2,4))

## the confidence intervals will be stored here

za=qnorm(0.95) ; n=lam=c(5,15,30,50) ; f=(n-1)/n

## f is used to get the unbiased variance estimator

m=sqrt(1/(2*pi)) ; s=1-1/(2*pi)

fixed4=(n^2*m^2+n*s)/za^2-n

## real values of Rosenthal's as fixed

random4=(lam^2*m^2+lam*(m^2+s))/za^2-lam

## real values of Rosenthal's as random

**coverage4**=matrix(nrow=4,ncol=6)

set.seed(123456)

## seed number

for (k in 1:4) {

for (i in 1:R) {

**z=rnorm(n[k])**

**## random values of z-statistics are generated**

**## When we draw from the half normal we use z=abs(rnorm(n[k],0,1)); when we draw from the skew normal distribution we use z=rsn(n[k],xi=0,omega=1,alpha=-0.5773503) or z=rsn(n[k],xi=0,omega=1,alpha=0.5773503) for negative and positive skewness resepctively ###**

**###############**

rosent4[i,k]=(sum(z)/za)^2-n[k]

## Rosenthal's value

m1=sqrt(1/(2*pi)) ; s1=1-1/(2*pi)

v4[i,1,k]=2*n[k]^2*s1*(2*n[k]*m1^2+s1)/za^4

## distributional variance of the fixed studies

m2=mean(z) ; s2=f[k]*var(z)

v4[i,2,k]=2*n[k]^2*s2*(2*n[k]*m2^2+s2)/za^4

## distributional variance of the random studies

V

4[i,3,k]=( (4*lam[k]^3+6*lam[k]^2+lam[k])*m1^4+(4*lam[k]^3+16*lam[k]^2+6*lam[k])*m1^2*s1+

(2*lam[k]^2+3*lam[k])*s1^2 )/za^4-2*( (2*lam[k]^2+lam[k])*m1^2+lam[k]*s1 )/za^2+lam[k]

## moments variance of the fixed studies

v4[i,4,k]=( (4*lam[k]^3+6*lam[k]^2+lam[k])*m2^4+(4*lam[k]^3+16*lam[k]^2+6*lam[k])*m2^2*s2+

(2*lam[k]^2+3*lam[k])*s2^2 )/za^4-2*( (2*lam[k]^2+lam[k])*m2^2+lam[k]*s2 )/za^2+lam[k]

## moments variance of the random studies

## then is the bootstrap case

t=rep(0,B)

for (j in 1:B) {

nu=sample(1:n[k],n[k],replace=T)

t[j]=(sum(z[nu])/za)^2-n[k] }

v4[i,5,k]=var(t)

mat16[i,,k]=c(rosent4[i,k]-1.96*sqrt(v4[i,1,k]),rosent4[i,k]+1.96*sqrt(v4[i,1,k]))

mat17[i,,k]=c(rosent4[i,k]-1.96*sqrt(v4[i,2,k]),rosent4[i,k]+1.96*sqrt(v4[i,2,k]))

mat18[i,,k]=c(rosent4[i,k]-1.96*sqrt(v4[i,3,k]),rosent4[i,k]+1.96*sqrt(v4[i,3,k]))

mat19[i,,k]=c(rosent4[i,k]-1.96*sqrt(v4[i,4,k]),rosent4[i,k]+1.96*sqrt(v4[i,4,k]))

mat20[i,,k]=c(rosent4[i,k]-1.96*sqrt(v4[i,5,k]),rosent4[i,k]+1.96*sqrt(v4[i,5,k])) } }

for (l in 1:4) coverage4[l,1]=1-(sum(mat16[,1,l]>fixed4[l])/R+sum(mat16[,2,l]<fixed4[l])/R)

for (l in 1:4) coverage4[l,2]=1-(sum(mat17[,1,l]>fixed4[l])/R+sum(mat17[,2,l]<fixed4[l])/R)

for (l in 1:4) coverage4[l,3]=1-(sum(mat18[,1,l]>random4[l])/R+sum(mat18[,2,l]<random4[l])/R)

for (l in 1:4) coverage4[l,4]=1-(sum(mat19[,1,l]>random4[l])/R+sum(mat19[,2,l]<random4[l])/R)

for (l in 1:4) coverage4[l,5]=1-(sum(mat20[,1,l]>fixed4[l])/R+sum(mat20[,2,l]<fixed4[l])/R)

for (l in 1:4) coverage4[l,6]=1-(sum(mat20[,1,l]>random4[l])/R+sum(mat20[,2,l]<random4[l])/R)

colnames(coverage4)=c('Dist_fixed','Mom_fixed','Dist_random','Mom_random',

'Boot_fixed', 'Boot_random')

rownames(coverage4)=c('n=5','n=15','n=30','n=50')

# **Meta-analyses Example Data**

Meta-analysis study by [[1](#_ENREF_1)]

| Authors | Year | z values |
| --- | --- | --- |
| Ligny | 1976 | 2.962859 |
| Adam | 1977 | 3.757467 |
| Gotz | 1979 | 1.650607 |
| Monteiro | 1981 | 2.166813 |
| Borgia | 1982 | 1.581611 |
| Frigerio | 1986 | 4.059215 |
| Surawicz | 1989 | 2.27334 |
| Wunderlich | 1989 | 1.487448 |
| Tankanow | 1990 | 0.17785 |
| Reid | 1992 | -0.0474 |
| McFarland | 1995 | 1.627997 |
| Lewis | 1998 | -0.79902 |
| Arvola | 1999 | 1.891838 |
| Benhamou | 1999 | -1.44082 |
| Vanderhoof | 1999 | 3.074713 |
| Felley | 2001 | -0.70246 |
| Thomas | 2001 | 0.210804 |
| Jirapinyo | 2002 | 1.553301 |
| Sheu | 2002 | 2.199867 |
| La Rosa | 2003 | 3.024602 |
| Sullivan | 2003 | -0.68994 |
| Erdeve | 2004 | 1.620978 |
| Erdeve | 2004 | 3.755796 |
| Lighthouse | 2004 | -0.70038 |
| Plummer | 2004 | 0 |
| Schrezenmeir | 2004 | -0.94973 |
| Tursi | 2004 | 1.864488 |
| Corrêa | 2005 | 2.198868 |
| Duman | 2005 | 2.608535 |
| Kotowska | 2005 | 3.186323 |
| Myllyluoma | 2005 | -0.90258 |
| Can | 2006 | 1.812663 |
| Beausoleil | 2007 | 1.969595 |
| Cindoruk | 2007 | 2.071372 |
| Conway | 2007 | 1.778388 |
| De Bortoli | 2007 | 2.914381 |
| Hickson | 2007 | 2.597848 |
| Park | 2007 | 2.95422 |
| Stein | 2007 | -0.98815 |
| Bravo | 2008 | 0.598887 |
| Kim | 2008 | -0.56565 |
| Koning | 2008 | 2.171299 |
| Ruszczynski | 2008 | -2.10516 |
| Safdar | 2008 | 1.256991 |
| Szymanski | 2008 | 0.595156 |
| Wenus | 2008 | 1.987948 |
| Engelbrektson | 2009 | -0.98909 |
| Merenstein | 2009 | 0.553913 |
| Szajewska | 2009 | 1.505209 |
| Gao | 2010 | 3.849341 |
| Koning | 2010 | -0.4195 |
| Li | 2010 | 2.816526 |
| Lönnermark | 2010 | -0.35165 |
| Sampalis | 2010 | 1.776439 |
| Song | 2010 | 0.426735 |
| Song | 2010 | 1.63705 |
| Yasar | 2010 | 0.249171 |
| Cimperman | 2011 | 1.533658 |
| de Vrese | 2011 | -0.35655 |
| Saneeyan | 2011 | 2.486708 |
| Selinger | 2011 | 1.630008 |
| Yoon | 2011 | -1.19603 |

Meta-analysis study by [[2](#_ENREF_2)]

| Authors | Year | Z |
| --- | --- | --- |
| Bell | 1989 | 0.829231 |
| Bochet | 2006 | 3.330265 |
| Bornstein | 2010a | 4.24916 |
| Sample b |  | 2.08 |
| Bornstein | 2010b | 4.55493 |
| Sample b |  | 2.702136 |
| Caldwell | 1976 | 2.688 |
| Camera | 2009 | 1.855267 |
| Carpenter | 2007b | 2.59759 |
| Sample b |  | 2.078072 |
| Sample c |  | 0.860488 |
| Carpenter | 2009 | 2.891 |
| Carpenter | 2004 | 3.72202 |
| Casari | 2009 | 6.465979 |
| Chen (Study 2) | 2009 | 1.803894 |
| Cinyabuguma | 2005 | 2.24 |
| Dickinson | 2001 | 5.145 |
| Sample b |  | 5.2675 |
| Dreber | 2008 | 4.131892 |
| Sample b |  | 0.824091 |
| Eek | 2002 | 7.115294 |
| Egas | 2008 | 0.696889 |
| Sample b |  | 4.355556 |
| Sample c |  | 1.829333 |
| Sample d |  | -0.80182 |
| Etran | 2009 | 3.223111 |
| Fehr | 2000 | 2.989831 |
| Sample b |  | 6.40396 |
| Fehr | 2002 | 14.7 |
| Fuster | 2010 | 3.477419 |
| Sample b |  | 3.411852 |
| Gachter | 2009 | 3.574118 |
| Sample b |  | 3.015385 |
| Sample c |  | -0.67586 |
| Sample d |  | -1.08138 |
| Gachter | 2011 | -0.98 |
| Sample b |  | 0.70359 |
| Sample c |  | 2.016 |
| Sample d |  | -2.0825 |
| Gachter | 2008 | 3.479 |
| Sample b |  | 8.131915 |
| Gachter | 2005 | 5.535878 |
| Sample a |  | 1.104225 |
| Sample b |  | 2.229725 |
| Herrmann | 2008 | 7.404444 |
| Study 2 |  | 7.410411 |
| Study 3 |  | 7.197377 |
| Study 4 |  | 8.563692 |
| Study 5 |  | 7.798298 |
| Study 6 |  | 8.949434 |
| Study 7 |  | 4.227451 |
| Study 8 |  | 0.522667 |
| Study 9 |  | 4.632727 |
| Study 10 |  | -1.04533 |
| Study 11 |  | 2.5872 |
| Study 12 |  | -1.03158 |
| Study 13 |  | -0.07127 |
| Study 14 |  | 9.740606 |
| Study 15 |  | 9.097358 |
| Study 16 |  | 7.423717 |
| Hopfensitz | 2009 | 3.441951 |
| Kieruj | 2008 | 7.466667 |
| Kocher | 2008 | 2.330811 |
| Komorita | 1985 | -0.70966 |
| Kroll | 2007 | 4.422564 |
| Martichuski | 1991 | 2.230345 |
| McCusker | 1995 | 4.174545 |
| Study 2 |  | 2.103415 |
| Mulder | 2005 | 3.5525 |
| Mulder | 2008 | 2.024615 |
| Mulder | 2001 | 2.232911 |
| Sample b |  | 2.184427 |
| Mulder | 2002 | 2.698182 |
| Mulder | 2003 | 1.905556 |
| Mulder | 2006a | 4.581818 |
| Study 2 |  | 3.705205 |
| Sample b |  | 1.737273 |
| Study 3 |  | 2.597 |
| Mulder | 2005 | 0.276056 |
| Mulder | 2006 | 2.94 |
| Myers | 2009 | 4.434747 |
| Nelissen | 2010 | 7.466667 |
| Nikiforakis | 2008 | 5.530959 |
| Sample b |  | 7.888395 |
| Sample c |  | 1.176 |
| Sample d |  | 6.055724 |
| Nikiforakis | 2010 | 3.364409 |
| Sample b |  | 3.82439 |
| Sample c |  | 5.115461 |
| Sample d |  | 5.371852 |
| O'Gorman | 2008 | 2.24918 |
| Sample b |  | -0.392 |
| O'Gorman | 2010 | 4.611765 |
| Sample b |  | 2.441967 |
| Page | 2005 | 6.653684 |
| Patel | 2010 | 0.658824 |
| Sample b |  | 0.897349 |
| Sample c |  | 0.722105 |
| Sample d |  | -1.68304 |
| Rand | 2009 | 3.486851 |
| Rapoport | 2001 | 1.448153 |
| Reuben | 2009 | 7.459578 |
| Sample b |  | 2.672727 |
| Sample c |  | 5.90481 |
| Sample d |  | 4.505455 |
| Riedl | 2009 | 1.26359 |
| Sample b |  | 1.180372 |
| Sample c |  | 1.801081 |
| Sample d |  | 0.969309 |
| Sato | 1987 | 4.856855 |
| Sefton | 2007 | 1.334468 |
| Sell | 1999 | 4.157576 |
| Shaw | 1976 | 2.655484 |
| Shinada | 2007 | 4.459 |
| Sample b |  | 2.512821 |
| Study 2 |  | 3.250732 |
| Sample b |  | 1.666 |
| Sutter | 2010 | 6.135652 |
| Sample b |  | 2.94 |
| Sutter | 2009 | 2.189091 |
| Tan | 2008 | 4.17088 |
| Tenbrunsel | 1999 | -1.93747 |
| Study 2 |  | -2.43185 |
| Study 3 |  | 1.583077 |
| Sample b |  | -0.96946 |
| Tyran | 2004 | 0 |
| van Prooijen | 2008 | -0.89091 |
| Sample b |  | -3.8357 |
| Study 2 |  | 0.598473 |
| Sample b |  | -2.24824 |
| Van Vugt | 1999 | 3.464186 |
| Walker | 2004 | -0.27509 |
| Sample b |  | 0.756491 |
| Wit | 1990 | 1.905047 |
| Study 2 |  | 0.368941 |
| Study 3 |  | 0 |
| Study 4 |  | 2.255849 |
| Xiao | 2010 | 1.2152 |
| Sample b |  | 3.01 |
| Sample c |  | 1.588276 |
| Sample d |  | 4.957647 |
| Sample e |  | 1.8424 |
| Sample f |  | 4.971707 |
| Sample g |  | 1.158957 |
| Sample h |  | 4.878222 |
| Yamagishi | 1986 | 3.275616 |
| Yamagishi | 1988 | 7.454426 |
| Yamagishi | 1992 | 5.377436 |

**References**

1. Hempel, S., et al., *Probiotics for the prevention and treatment of antibiotic-associated diarrhea: a systematic review and meta-analysis.* JAMA, 2012. **307**(18): p. 1959-69.

2. Balliet, D., L.B. Mulder, and P.A. Van Lange, *Reward, punishment, and cooperation: a meta-analysis.* Psychological Bulletin, 2011. **137**(4): p. 594-615.
